# 3D DXA Hip Differences in Patients with Acromegaly or Adult Growth Hormone Deficiency

**DOI:** 10.3390/jcm10040657

**Published:** 2021-02-09

**Authors:** Luis Gracia-Marco, Sheila Gonzalez-Salvatierra, Antonia Garcia-Martin, Esther Ubago-Guisado, Beatriz Garcia-Fontana, José Juan Gil-Cosano, Manuel Muñoz-Torres

**Affiliations:** 1 PROFITH “PROmoting FITness and Health Through Physical Activity” Research Group, Sport and Health University Research Institute (iMUDS), Department of Physical and Sports Education, Faculty of Sport Sciences, University of Granada, Camino de Alfacar 21, 18071 Granada, Spain; lgracia@ugr.es (L.G.-M.); josejuangil@ugr.es (J.J.G.-C.); 2 Instituto de Investigación Biosanitaria ibs. GRANADA, 18012 Granada, Spain; 3 Bone Metabolic Unit, Endocrinology and Nutrition Division, University Hospital Clínico San Cecilio, Instituto de Investigación Biosanitaria de Granada (Ibs.GRANADA), Av. de la Ilustración s/n, 18016 Granada, Spain; sgsalvatierra@ugr.es (S.G.-S.); antoniagarciamartin@gmail.com (A.G.-M.); bgfontana@fibao.es (B.G.-F.); mmt@mamuto.es (M.M.-T.); 4 Department of Medicine, University of Granada, Av. de la Investigación 11, 18016 Granada, Spain; 5 CIBERFES, Instituto de Salud Carlos III, C/Sinesio Delgado 4, 28029 Madrid, Spain; 6 Escuela Andaluza de Salud Pública (EASP), 18011 Granada, Spain

**Keywords:** DXA, bone modelling and remodelling, bone QCT/microCT, osteoporosis

## Abstract

The skeleton is regulated by and responds to pituitary hormones, especially when the circulating levels are perturbed in disease. This study aims to analyse the between-group differences in 3D dual-energy X-ray absorptiometry (DXA) parameters at the hip site among patients with acromegaly or adult growth hormone deficiency (AGHD) and a healthy control group. The current cross-sectional study includes data for 67 adults, 20 with acromegaly, 14 with AGHD and 33 healthy controls. We obtained the areal bone mineral density (aBMD) outcomes using DXA and cortical and trabecular parameters using 3D-DXA software (3D-SHAPER). The mean-adjusted 3D-DXA parameters did not differ between acromegaly patients and the controls (*p* > 0.05); however, we found cortical bone impairment (−7.3% to −8.4%; effect size (ES) = 0.78) in AGHD patients (*p* < 0.05). Differences in the cortical bone parameters were more evident when comparing AGHD patients (−8.5% to −16.2%; ES = 1.22 to 1.24) with acromegaly patients (*p* < 0.05). In brief, the 3D mapping highlighted the trochanter as the site with greater cortical bone differences between acromegaly patients and the controls. Overall, AGHD patients displayed lower cortical parameters at the trochanter, femoral neck and intertrochanter compared to the controls and acromegaly patients. To sum up, 3D-DXA provided useful information about the characteristics of bone involvement in growth hormone (GH)-related disorders. Patients with AGHD showed distinct involvement of the cortical structure.

## 1. Introduction

Growth hormone (GH) and insulin-like growth factor 1 (IGF1) are classically considered anabolic hormones that stimulate bone modelling and remodelling. Their main effects are the stimulation of osteoblastogenesis and the activation of mature osteoblasts, contributing to longitudinal growth [1].

Patients with acromegaly present excessive levels of GH and IGF-I and characteristically enlarged bones [1]. Previous research showed acromegaly to have a site-specific effect on the areal bone mineral density (aBMD). In this regard, findings from a meta-analysis [2] showed no effect on the aBMD at the lumbar spine but a positive effect at the total hip and femoral neck sites. A recent review highlighted that GH hypersecretion is linked to bone microstructure deterioration, and therefore, patients with acromegaly are more prone to develop vertebral fractures, which could be related to other comorbidities such as hypogonadism [3]. Previous studies reported an abnormal cortical bone microstructure in acromegaly patients with vertebral fractures [4,5]. Using high-resolution peripheral quantitative computed tomography (HRpQCT), Silva et al. [6] and Madeira et al. [7] reported that patients with acromegaly had an impaired trabecular microstructure of the radius and distal tibia, respectively. Silva et al. [6] found that patients with acromegaly had greater cortical thickness, cortical area and cortical porosity at the radius compared to controls, while no differences were found at the tibia. GH deficiency (GHD) is a pituitary defect of importance in the clinical picture of hypopituitarism [8]. Skeletal fragility is frequently found in adult patients with GHD [8], and so are impaired aBMD and volumetric BMD (vBMD) [9,10]. Recently, in childhood-onset GHD adults, Yang et al. [11] observed decreased trabecular and cortical bone parameters of both the tibia and the radius using HRpQCT. However, a recent study in adult males with GHD did not find abnormal bone features in the trabecular and cortical microarchitecture using the same technique [6].

Dual-energy X-ray absorptiometry (DXA) is considered a reference (non-invasive) method for assessing aBMD in clinical settings, and its use has spread worldwide in numerous studies with different population groups [12]. Due to its limited resolving power, DXA cannot distinguish between cortical and trabecular bone [13]. Techniques like pQCT or HRpQCT enable in vivo assessment of the trabecular and cortical bone microarchitecture, but the high costs represent a barrier for most laboratories. 

Therefore, an alternative in clinical settings could arise from the combination of aBMD data (from DXA) with 3D QCT-like parameters of cortical and trabecular bone. In this report, we use aBMD of the hip site and a recently developed 3D-DXA software algorithm to quantify the vBMD of the cortical and trabecular bone compartments as well as the cortical thickness, among other parameters. The 3D-DXA models and measurements were compared against QCT [14], and the resulting data suggest that it could be a feasible alternative in the absence of pQCT [15].

Thus, the aim of this study is to examine the aBMD and 3D-DXA differences at the hip site among patients with acromegaly or GHD and a healthy control group.

## 2. Materials and Methods

### 2.1. Study Design and Participants

The current cross-sectional study includes data from 67 adults (51 females) from Granada (Spain). Twenty patients with acromegaly, 14 patients with adult GHD (AGHD) and 33 healthy controls were included. The proportion of premenopausal/postmenopausal women did not vary among the groups. The diagnosis of acromegaly was established based on the clinical characteristics, high levels of GH that were not suppressible following an oral glucose tolerance test, and IGF-1 levels exceeding the upper limit of the normal range. Taking into account the GH (<1 ng/mL) and IGF-I (within the normal range) levels at the time of the study, the patients were considered active (*n* = 3) or controlled (*n* = 17). Sensitivity analyses excluding patients with active acromegaly (*n* = 3) were performed.

AGHD patients were diagnosed after the age of 18 years as a consequence of hypothalamic–pituitary diseases. The diagnosis of GHD was confirmed by the insulin tolerance test and the glucagon test following the recommendations of the Spanish National Health Service. A serum GH value of <3 ng/mL at any time during the testing was chosen as a criterion for the presence of GHD, following the consensus guidelines for the diagnosis and treatment of adults with GHD [16]. The pituitary pathology of AGHD patients was surgery and radiotherapy for pituitary tumours for six patients, pituitary apoplexy for three, idiopathic panhypopituitarism for two, craniopharyngioma for one and histiocytosis X for one. At the time of the study, 11 (out of 14) patients were on GH treatment, and sensitivity analyses excluding those who were not undergoing treatment (*n* = 3) were performed. In all patients, concomitant pituitary deficiencies were treated with stable replacement therapy.

Healthy controls with a chronologic age within 5 years from that of the patients with acromegaly or AGHD were also recruited. At the moment of recruitment, none of the participants presented diseases known to affect bone metabolism. Early menopause, a history of previous fragility fracture and the use of bisphosphonates, glucocorticoids or other antiosteoporosis treatment in the two years before inclusion were considered exclusion criteria. The data were obtained in 2019 at the Endocrinology Unit of the University University Hospital Clínico San Cecilio of Granada.

The study was granted ethical approval by the ethics committee of the Provincial Biomedical Research of Granada and followed the Declaration of Helsinki. We obtained informed consent from all participants.

### 2.2. Outcome Measures

#### 2.2.1. Areal Bone Mineral Density

The non-dominant hip was scanned with a Hologic QDR 4500 densitometer (Hologic Series Discovery QDR, Bedford, MA, USA). The aBMD measurements of the total hip, trochanter and femoral neck were obtained. A trained operator performed all DXA scans and analyses according to the recommendations from the International Society of Clinical Densitometry [17]. A spine phantom was used to calibrate the DXA scans on a daily basis. The coefficients of variation in our laboratory were 1.5% and 1.8% for the total hip and femoral neck, respectively [18].

#### 2.2.2. 3D-DXA Modelling

We used 3D-Shaper software (version 2.2, Galgo Medical, Barcelona, Spain) to assess the cortex, femoral shape and trabecular macrostructure from DXA scans of the acromegaly, AGHD and control groups. The full details of the modelling method used in the software have been published previously [14]. The vBMD (mg/cm^3^) values (of the cortical, trabecular and integral bone compartments) of the total femur and cortical thickness (mm) were computed [19]. The trabecular vBMD (mg/cm^3^) measures the density of the trabecular compartment. 

The integral compartment is measured by the integral vBMD, which is the union of the cortical and trabecular compartments. The correlations between 3D-DXA and QCT for the integral vBMD, cortical vBMD, trabecular vBMD and cortical thickness were 0.95, 0.93, 0.86 and 0.91, respectively [14]. The coefficients of variation for the cortical surface BMD (sBMD), cortical vBMD, cortical thickness and trabecular vBMD, and cortical vBMD and cortical sBMD were 1.5%, 1.7%, 4.5% and 1.5%, respectively [15].

#### 2.2.3. Anthropometric Measures

The weight (kg) was obtained with an electronic scale (SECA 861 and 760, Hamburg, Germany). The height (cm) was obtained using a stadiometer (SECA 225 and 220, Hamburg, Germany). The body mass index (BMI) was computed as the ratio between the weight (kg) and squared height (m^2^).

#### 2.2.4. Serum Measurements

Venous blood samples were obtained by venipuncture after an overnight fast and immediately kept frozen at −80 °C. The serum levels of GH and IGF-I were measured using commercial two-site chemiluminiscent immunoassays (Immulite^®^ 2000 XPi, Siemens, Los Angeles, CA, USA).

### 2.3. Statistical Analysis

We used SPSS IBM statistics (version 20 for Windows, Chicago, IL, USA) to analyse the data, and the alpha level was set at 5%. The distribution of variables was checked using the Kolmogorov–Smirnov test, as well as a visual check of the histograms, skewness and kurtosis values, and Q-Q and box plots. Descriptive analyses of the continuous variables were performed by analysis of variance (ANOVA) or the chi-square test (for categorical variables). Analysis of covariance (ANCOVA) was used to examine differences in the outcome variables (aBMD and 3D-DXA parameters) between the acromegaly, AGHD and control groups (percentage of difference is also provided). Sex, age and the BMI were used as covariates. The Sidak correction was used for adjusted pairwise testing. The effect sizes (ESs, Cohen’s *d*) are shown, and the interpretation is 0.2 (small), 0.5 (medium) and 0.8 (large) [20]. The 3D spatial distribution of the differences (acromegaly vs. control, AGHD vs. control and AGHD vs. acromegaly) in the cortical bone (cortical sBMD, cortical vBMD and cortical thickness) were computed and are shown.

## 3. Results

Table 1 shows the descriptive characteristics of the participants in the acromegaly, AGHD and control groups. Overall, the proportions of males (21.4%–25.0%) and females (75.0%–78.6%) were similar across the groups, and no significant differences were found in age, weight and the BMI (all *p* > 0.05). The time since diagnosis was 10 ± 7 years in patients with acromegaly and 22 ± 10 years in AGHD patients. The duration of GH replacement therapy in patients with AGHD was 19 ± 7 years. Crude aBMD and 3D-DXA parameters of the cortical sBMD and cortical vBMD significantly differed among the groups (all *p* < 0.05).

**Figure 1:**
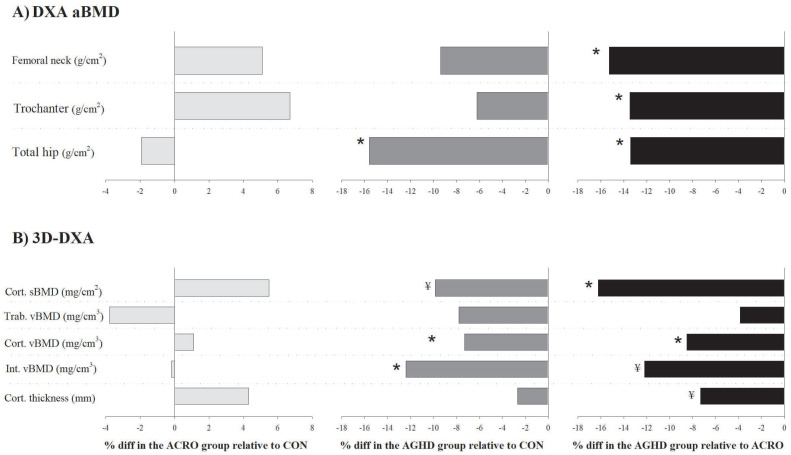
Adjusted differences (%) (by age, sex and body mass index) in (**A**) the areal bone mineral density (aBMD, g/cm^2^) and (**B**) 3D-DXA parameters between patients with acromegaly (*n* = 20), adult growth hormone deficiency (AGHD, *n* = 14) and the healthy control group (*n* = 33). * *p* < 0.05 and ^¥^ *p* < 0.09 relative to the control group.

Table 2 shows mean-adjusted (by age, sex and BMI) differences in the aBMD and 3D-DXA parameters among the acromegaly, AGHD and control groups (percentage of differences are shown in Figure 1). No significant differences in the aBMD were observed between the acromegaly and control groups (all *p* > 0.05). The aBMD was significantly lower in the AGHD group compared to the control group at the total hip (−15.6%, *p* = 0.002, ES = 1.13) and compared to the acromegaly group at all sites: femoral neck (−15.3%, *p* = 0.020, ES = 0.99), trochanter (−13.5%, *p* = 0.015, ES = 1.01) and total hip (−13.4%, *p* = 0.018, ES = 0.99). The 3D-DXA parameters did not differ between the acromegaly and control groups (all *p* > 0.05). Lower cortical vBMD (−7.3%, *p* = 0.004, ES = 0.78) and integral vBMD (−8.4%, *p* = 0.048, ES = 0.78) were identified in the AGHD group compared to the control group. The cortical sBMD (−16.2%, *p* = 0.003, ES = 1.22) and cortical vBMD (−8.5%, *p* = 0.002, ES = 1.24) were also lower in the AGHD group compared to the acromegaly group. 

Considerable trends towards significance were found for the cortical sBMD (−9.9%, *p* = 0.071, ES = 0.72) between the AGHD and control groups and for the integral vBMD (−12.2%, *p* = 0.087, ES = 0.78) and cortical thickness (−7.3%, *p* = 0.066, ES = 0.82) between the AGHD and acromegaly groups. We performed sensitivity analyses excluding patients with active acromegaly (*n* = 3) and those not undergoing GH treatment (*n* = 3) and found similar results. In brief, AGHD patients had impaired cortical vBMD compared to the controls (*p* = 0.027). They also had a lower cortical vBMD (*p* = 0.011) and cortical sBMD (*p* = 0.036) compared to patients with acromegaly.

**Figure 2:**
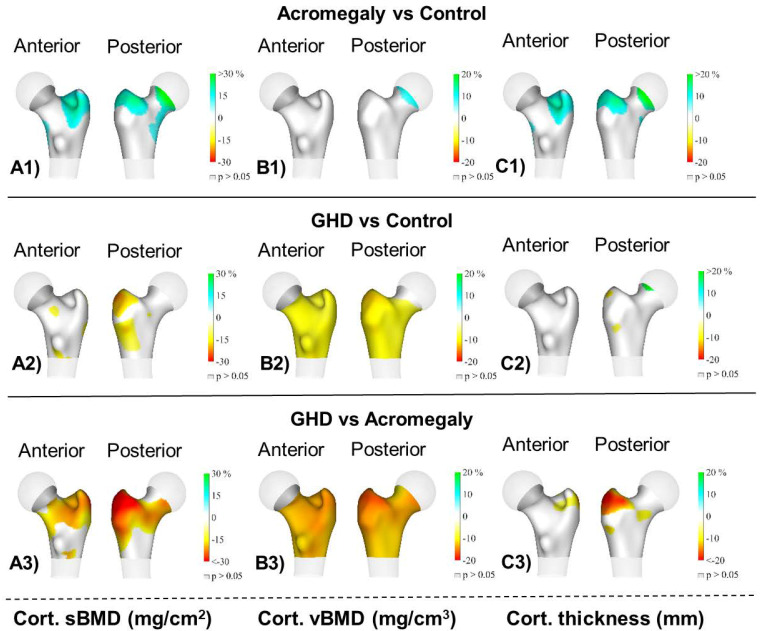
Distribution of the average differences (*p* < 0.05) in (**A1**–**A3**) the cortical surface bone mineral density (cortical sBMD), (**B1**–**B3**) cortical volumetric bone mineral density (cortical vBMD) and (**C1**–**C3**) cortical thickness of the total femur region among groups: acromegaly (*n* = 20), adult growth hormone deficiency (AGHD, *n* = 14) and the healthy control group (*n* = 33). Regions with no significant differences are coloured in grey (*p* > 0.05).

Figure 2 shows the 3D mapping of differences in the cortical compartment among the groups. The main findings showed that the acromegaly group had a significantly higher cortical sBMD and cortical thickness at the trochanter (*p* < 0.05) compared to the control group. The AGHD group had a lower cortical sBMD and cortical vBMD at the femoral neck and trochanter and also a lower cortical vBMD at the intertrochanter (all *p* < 0.05) compared to the control group. Finally, the AGHD group had a lower cortical sBMD at the trochanter; a lower cortical vBMD at the femoral neck, trochanter and intertrochanter; and a lower cortical thickness at the trochanter (all *p* < 0.05) compared to the acromegaly group.

## 4. Discussion

This is the first study combining hip DXA-derived and 3D-DXA outcomes of cortical and trabecular bone in AGHD patients. This is also the first study comparing bone differences between acromegaly patients, AGHD patients and healthy controls. The main findings showed that (1) acromegaly patients and healthy controls did not differ in the aBMD or 3D-DXA parameters, (2) AGHD patients presented a poorer total hip aBMD and cortical bone parameters (cortical vBMD and integral vBMD) compared to the control group, (3) AGHD patients had al ower aBMD at all measured sites and cortical bone (cortical sBMD and cortical vBMD) compared to acromegaly patients and (4) the hip trabecular bone did not differ among groups.

### 4.1. Differences in aBMD among Groups

The majority of the effects of GH on bone metabolism are mediated by the anabolic hormone IGF-I (produced systemic and locally) by increasing osteoblast differentiation [21]. In acromegaly, aBMD overestimations may occur, particularly at the lumbar spine, due to the joint degenerative disorders that appear as a consequence of GH hypersecretion [22]. In contrast, the aBMD may be underestimated due to the bone enlargement caused by excess GH [23]. Despite this, Mazziotti et al. showed, in their meta-analysis, a huge heterogeneity of literature data, showing normal or increased aBMD in acromegaly [2], and only a few studies have shown the opposite [24,25]. In our study, the aBMD did not differ between acromegaly patients and healthy controls, which seems to agree with the findings of the previous meta-analysis and also those recently published by Silva et al. [6].

GHD is a rare and important disorder in the clinical presentation of hypopituitarism. Previous research showed reduced bone turnover in AGHD patients [26]. Our AGHD patients were not diagnosed in childhood, which may predispose patients to more pronounced bone loss due to the longer duration of the disease [27,28,29] and therefore compromise peak bone mass acquisition [30,31]; however, they had a significantly lower aBMD compared to both control and acromegaly groups. The differences in the aBMD between AGHD patients and controls were only evident for the total hip, not for the femoral neck or trochanter sites. 

The lack of consistent significant differences in the aBMD outcomes could be explained by the age of our AGHD participants (57.4 ± 12.3 years). Previous research showed age as a key factor affecting the degree of aBMD loss in AGHD. For example, more severe osteopenia was observed in patients <30 years of age [32,33], whereas similar aBMD values were observed in patients >55 years of age when compared to healthy controls [32,34]. Of our AGHD patients, 78.5% were under GH treatment, which might also explain why the aBMD only differed in one out of the three sites examined in this study.

In our study, the main differences in the aBMD were between AGHD and acromegaly groups. The aBMD was lower in AGHD patients compared to acromegaly patients at all measured sites of the hip, with differences ranging from −13.4% to −15.3%. These findings support the anabolic role of GH and IGF-I and their contribution to bone formation [1]. Previous research showed GHD to be accompanied by abnormalities in the circadian rhythm of parathyroid hormone, and this might affect bone remodelling [35]. Mazzioti et al. demonstrated, in their meta-analysis, that hypogonadism contributes to a lower aBMD at the femoral neck in acromegaly [2]. 

In our study, the percentage of participants with hypogonadism was higher in the AGHD than the acromegaly group (87.5 vs. 30.0%), which could also explain some of the differences in the aBMD between groups. Finally, our findings are reinforced by the fact that the AGHD and acromegaly groups were similar in terms of age and sex. As expected, there were differences between the groups in height (*p* = 0.017), although the differences in body mass did not reach statistical significance (*p* = 0.112). To overcome this and also to control for the possible intra-group differences, we took into account age, sex and te BMI (which includes height and body mass in the calculation) as covariates.

### 4.2. Cortical and Trabecular Bone Differences Assessed by 3D-DXA

Previous research showed a controversial effect of excess GH/IGF-I on trabecular and cortical bone in acromegaly [36]. Kuzma et al. [37] published the first study using 3D-SHAPER software in acromegaly and found that regardless of the presence of vertebral fractures, acromegaly patients had less trabecular bone, while cortical density was only decreased in those with vertebral fractures. In our study, we did not find trabecular bone differences between acromegaly patients and the controls. We did not find cortical bone differences between these groups either, which could be (partially) explained because we only had one patient with a history of vertebral fractures, and all patients were on stable replacement therapy. 

Malgo et al. observed a lower bone material strength index (measured with impact micro-indentation at the midshaft of the tibia) in acromegaly patients compared to controls with a comparable age and femoral neck aBMD, suggesting impaired cortical bone in acromegaly [38]. In contrast, Madeira et al. showed increased cortical thickness in the distal radius in acromegaly in comparison with a control group using HRpQCT [7]. In our study, while the cortical bone appeared to be higher in acromegaly patients compared to healthy controls, the differences did not reach statistical significance. As an example, the cortical thickness was 4.3% higher (non-significant) in acromegaly patients compared to the controls. We did not measure the bone microarchitecture at the radius; however, it is possible that weight bearing (in the femur) may limit the differences in the cortical thickness (and other cortical parameters).

Our data revealed impaired cortical bone in AGHD patients compared to the controls, mainly for the cortical and integral vBMD (−7.3% and −8.3%, respectively). We also observed a trend for a lower sBMD, a highly accurate indicator of the cortical bone strength [39], in AGHD patients. In this line, Bravenboer et al. observed a decreased bone formation rate, as well as osteoid and mineralising surfaces from bone biopsies obtained from GHD men [40]. 

Yang et al. [11] found impaired cortical and trabecular vBMD, bone microarchitecture and estimated bone strength in childhood-onset AGHD using HRpQCT. In contrast, Silva et al. did not find significant differences in the estimated bone strength and cortical bone microarchitecture between patients with GHD and controls using microfinite analyses and HRpQCT. However, some of the patients were treated with GH replacement 12 months before the study, and this could counteract the (expected) cortical bone impairment at the time of testing [6].

In our study, we observed cortical bone impairment in AGHD patients in comparison to acromegaly patients. The AGHD patients displayed a lower cortical sBMD (−16.2%) and cortical vBMD (−8.5%), while the differences in the integral vBMD and cortical thickness were trendy (*p* = 0.087 and *p* = 0.066, respectively). Our findings agree with those reported by Silva et al., who found impaired cortical bone microarchitecture in GHD patients compared to acromegaly patients [19]. Previous evidence assessing lumbar spine outcomes, a site with predominant trabecular bone, suggested that the trabecular bone microstructure was affected after being exposed to pathologically high levels of GH and IGF-I [1]. This lowers the bone strength and contributes to the increased risk of vertebral fractures in patients with acromegaly [3]. 

Silva et al. found decreased trabecular bone microarchitecture of the radius (also a site with predominant trabecular bone) in acromegaly patients compared to the healthy control group using HRpQCT [6]. Madeira et al. showed that eugonadal acromegalic patients had impaired trabecular bone at the radius and distal tibia using HRpQCT. Interestingly, we did not observe significant differences in the trabecular bone of the femoral neck in any of the group comparisons. Non-significant, trabecular vBMD appeared to be slightly impaired in both the AGHD and acromegaly groups in comparison to the healthy control group (−7.8% and −3.8%, respectively). Similarly, the trabecular vBMD was slightly lower (−3.9%, non-significant) in AGHD patients compared to acromegaly patients.

Despite our analysis being adjusted by age, sex and the BMI, these comparisons must be interpreted with caution due to differences in the measurement site, the predominance of cortical/trabecular bone at that site and also the patient’s characteristics (30% of our acromegaly patients and 87.5% of our AGHD patients presented with hypogonadism). In this regard, our AGHD and acromegaly groups represent day-to-day patients in clinical practice, with a combination of active/controlled patients with acromegaly and replaced/unreplaced GHD. We performed sensitivity analyses excluding patients with active acromegaly (*n* = 3) and also those with unreplaced GHD and found very similar results, suggesting impaired cortical vBMD in the AGHD group compared to the control group and a lower cortical vBMD and cortical sBMD in AGHD patients compared to acromegaly patients.

Our 3D mapping showed differences in the cortical sBMD among groups mainly at the trochanter site. The impaired cortical vBMD observed in AGHD patients in comparison to both the control and acromegaly groups affected the femoral neck and trochanteric and intertrochanteric sites. Lastly, the cortical thickness appeared to be similar in AGHD patients and the controls; however, the acromegaly patients displayed thicker cortical bone at the trochanter. 

These novel findings are of relevance since lower bone strength increases the vulnerability to fracture [41], and fractures in the trochanter are related to up to twice the short-term mortality of cervical fractures in the elderly [42]. Cortical bone plays a key role in the axial load-bearing capacity of long bones and, therefore, decreases in the cortical vBMD and cortical thickness are considered proxy markers for bone loss [43]. More specifically, the evaluation of cortical thinning (with X-rays) identified 21% additional fracture cases over those identified by a T-score < −2.5 [43]. This underlines the importance of evaluating cortical bone and investigating new methodologies that improve the clinical diagnosis in conditions in which the aBMD presents limitations, such as acromegaly and AGHD [23]. These findings also support an increased or decreased site-specific fracture risk, depending on the condition.

Our findings suggest that combining the aBMD with 3D-DXA parameters may be a good approach for clinicians to assess bone characteristics in day-to-day practice at the hip site, especially in patients with AGHD. Of note, 11 (out of 14) AGHD patients had been on treatment for 19 (7) years; however, bone impairment was still evident. We did not find significant correlations between bone outcomes and disease duration in treated GHD patients. This is of importance because other techniques, such as HRpQCT, are only available in a few places worldwide and are unlikely to become widely used [44].

### 4.3. Strengths and Limitations

This is the first study reporting cortical and trabecular 3D-DXA outcomes and quantifying the differences among acromegaly patients, AGHD patients and healthy controls. The participants were very similar in terms of age and sex. As limitations, the cross-sectional design precludes any determination of causality in our findings. The sample size was relatively small but in line with those of previous studies [6]. This might explain the borderline differences among groups found in some of the cortical bone-related outcomes. The low prevalence of both diseases makes it very challenging to carry out a study with a large sample size and difficult to establish a real impact of the underlying pathology.

## 5. Conclusions

The main findings of this study suggest a similar bone status between acromegaly patients and healthy controls but impaired cortical (but not trabecular) bone in AGHD patients compared to both other groups. Future longitudinal studies with greater sample sizes are warranted to confirm the potential of 3D-DXA in clinical settings.

## Figures and Tables

**Table 1:** Descriptive characteristics of the study groups.

	CON (*n* = 33)		ACRO (*n* = 20)		AGHD (*n* = 14)		Overall *p*-Value
Sex (*n*,%) Male	8, 24.2		5, 25.0		3, 21.4		0.969
Female	25, 75.8		15, 75.0		11, 78.6		
Hypogonadism (*n*,%)	N/A		6, 30.0		12, 87.5		-
Prevalent fracture (*n*,%)	N/A		1, 5.0		2, 14.3		-
Smoking (*n*,%)	7, 21.2		5, 25.0		5, 35.7		0.579
	**Mean**	**SD**	**Mean**	**SD**	**Mean**	**SD**	
Age (years)	56.1	12.0	54.8	11.5	57.4	12.3	0.816
Duration of disease (years)	N/A		10	7	22	10	-
Duration of GH replacement therapy (years)	N/A		-	-	19	7	-
Height (m)	1.62	0.10	1.65	0.08	1.56	0.09	**0.020**
Weight (kg)	72.4	11.7	78.5	16.5	68.9	10.2	0.096
BMI (kg/m^2^)	27.7	4.6	28.8	5.0	28.6	5.2	0.711
IGF-I (ng/mL)	N/A		191.2	141.7	145	39.3	-
GH (ng/mL)	N/A		1.65	2.0	0.72	0.78	-
aBMD							
Femoral neck (g/cm^2^)	0.779	0.137	0.833	0.161	0.705	0.117	**0.040**
Femoral neck T-score	−0.855	1.001	−0.320	1.258	−1.400	0.981	**0.020**
Trochanter (g/cm^2^)	0.682	0.099	0.741	0.127	0.642	0.100	**0.031**
Total hip (g/cm^2^)	0.953	0.124	0.950	0.156	0.823	0.142	**0.011**
3D-DXA							
Cortical sBMD (mg/cm^2^)	163.32	20.28	175.75	31.53	148.86	22.80	**0.010**
Trabecular vBMD (mg/cm^3^)	183.40	36.44	181.97	57.41	169.18	41.98	0.594
Cortical vBMD (mg/cm^3^)	836.14	59.04	851.96	69.97	779.79	45.55	**0.003**
Integral vBMD (mg/cm^3^)	327.83	47.14	334.72	68.10	291.27	54.41	0.066
Cortical thickness (mm)	1.95	0.16	2.06	0.25	1.90	0.21	0.060

Values shown as the mean and standard deviation (SD), or percentages. Bold letters denote significant differences (*p* < 0.05). Groups: CON, control; ACRO, acromegaly; AGHD, adult growth hormone deficiency. Abbreviations: BMI, body mass index; IGF-I, insulin-like growth factor I; GH, growth hormone; aBMD, areal bone mineral density; sBMD, surface bone mineral density; vBMD, volumetric bone mineral density; 3D-DXA: three-dimension dual-energy X-ray absorptiometry N/A: not applicable.

**Table 2:** Differences (adjusted by age, sex and body mass index) in the areal bone mineral density (aBMD) and 3D-DXA parameters between the study groups.

	CON (*n* = 33)		ACRO (*n* = 20)		AGHD (*n* = 14)			Pairwise *p*-Value (Effect Size) *		
	Mean	SE	Mean	SE	Mean	SE	Overall *p*-Value	CON-ACRO	CON-AGHD	AGHD-ACRO
aBMD										
Femoral neck (g/cm^2^)	0.781	0.019	0.823	0.025	0.714	0.029	**0.024**	NS (0.36)	NS (0.63)	0.020 (0.99) *
Trochanter (g/cm^2^)	0.685	0.015	0.732	0.019	0.645	0.023	**0.016**	NS (0.57)	NS (0.43)	0.015 (1.01) *
Total hip (g/cm^2^)	0.957	0.020	0.939	0.025	0.828	0.030	**0.002**	NS (0.13)	**0.002 (1.13)**	0.018 (0.99) *
3D-DXA										
Cortical sBMD (mg/cm^2^)	164.19	3.43	173.74	4.41	149.467	5.26	**0.004**	NS (0.50)	0.071 (0.72) ^¥^	0.003 (1.22) *
Trabecular vBMD (mg/cm^3^)	184.81	6.51	178.07	8.38	171.44	9.90	0.518	NS (0.18)	NS (0.36)	NS (0.18)
Cortical vBMD (mg/cm^3^)	838.16	9.35	847.66	12.03	781.16	14.34	**0.002**	NS (0.19)	0.004 (1.05) *	0.002 (1.24) *
Integral vBMD (mg/cm^3^)	330.05	8.04	329.42	10.35	293.60	12.33	**0.040**	NS (0.00)	0.048 (0.78) *	0.087 (0.78) ^¥^
Cortical thickness (mm)	1.955	0.030	2.043	0.038	1.904	0.045	0.057	NS (0.54)	NS (0.28)	0.066 (0.82) ^¥^

Values presented as mean and standard error (SE). Bold letters denote a significant difference (*p* < 0.05). In addition, near-significant differences (*p* < 0.08) between groups are depicted by ^¥^. Groups: CON, control; ACRO, acromegaly; AGHD, adult growth hormone deficiency. Abbreviations: aBMD, areal bone mineral density; sBMD, surface bone mineral density; vBMD, volumetric bone mineral density; NS: not significant. * The interpretation of the effect size (ES) is 0.2 (small), 0.5 (medium) and 0.8 (large).

## Data Availability

The data presented in this study are available upon request from the corresponding author. The data are not publicly available due to ethical constraints.
